# Nonoperative Modalities to Treat Symptomatic Cervical Spondylosis

**DOI:** 10.1155/2012/294857

**Published:** 2011-08-01

**Authors:** Kieran Michael Hirpara, Joseph S. Butler, Roisin T. Dolan, John M. O'Byrne, Ashley R. Poynton

**Affiliations:** ^1^Cappagh National Orthopaedic Hospital, Finglas, Dublin 11, Ireland; ^2^Waterford Regional Hospital, Waterford, Ireland

## Abstract

Cervical spondylosis is a common and disabling condition. It is generally felt that the initial management should be nonoperative, and these modalities include physiotherapy, analgesia and selective nerve root injections. Surgery should be reserved for moderate to severe myelopathy patients who have failed a period of conservative treatment and patients whose symptoms are not adequately controlled by nonoperative means. A review of the literature supporting various modalities of conservative management is presented, and it is concluded that although effective, nonoperative treatment is labour intensive, requiring regular review and careful selection of medications and physical therapy on a case by case basis.

## 1. Introduction

Spondylosis refers to age-related degenerative changes within the spinal column [1], which in the cervical spine may be asymptomatic or can present as pure axial neck pain, cervical radiculopathy, cervical myelopathy, or cervical myeloradiculopathy. Radiological evidence of asymptomatic cervical spondylosis is seen frequently [1, 2], with an incidence of 50% over the age of 40 and 85% over the age of sixty [2, 3]. Unfortunately, neck pain and radiculopathy are relatively common, with about two thirds of the UK population having neck pain at some point in their lives [4, 5], and 34% of responders in a Norwegian survey of 10,000 adults having experienced neck pain in the previous year [6]. Nonsurgical treatment is usually the most appropriate course of initial management [7, 8], with operative intervention being reserved for moderate to severe myelopathy, or cases with unremitting and progressive symptoms that have failed medical treatment [7, 9, 10]. Despite the high incidence of symptomatic cervical degeneration and the widespread use of nonoperative techniques to treat this condition, the number of comparative trials in the literature is small and usually of poor quality [11]. In this paper we attempt to summarise the recommendations of the literature with regards to treating symptomatic cervical spondylosis, including cervical radiculopathy and mild myelopathy.

## 2. Pathophysiology

### 2.1. Neck Pain

In the vast majority of patients axial neck pain is related to muscular and ligamentous factors related to injury, poor posture, stress, or chronic muscle fatigue [12]. The cervical discs and facet joint scan are also sources of pain secondary to degenerative disease. The pathway whereby a degenerate disc causes neck pain is disputed, however the nerve supply to the peripheral portion of the intervertebral disc may be responsible for the direct sensation of pain [13, 14]. The sinuvertebral nerve is formed from branches of the ventral nerve root and the sympathetic plexus [14] and supplies nerve endings to the annulus, posterior longitudinal ligament, periosteum of the vertebral body and pedicle, and the adjacent epidural veins. Direct stimulation of each disc by discography produces reliable patterns of pain [15], and this would suggest that the degeneration of the disc may be responsible for axial neck pain. Provocative injection of the facet joint also produces reliable patterns of axial neck pain and pain in the shoulder girdle [16], indicating that the facet joint may also be responsible for axial neck pain. This is further confirmed when pain is removed by injection of anaesthetic into the facet joint [17] around the dorsal primary rami [18]. Degeneration of the upper cervical joints can cause severe suboccipital pain radiating to the neck or ear, and injection of the atlanto-occipital and atlantoaxial joints results in a reproducible pain pattern in this region [19].

### 2.2. Cervical Radiculopathy and Myelopathy

Age-related degeneration of the intervertebral disc results in loss of viscoelasticity and a loss of height with associated posterior bulging. As the disc height decreases the ligamentum flavum folds, as does the facet joint capsule, causing a decrease in the dimensions of the canal and exit foramina. Osteophytes form posteriorly around the disc, and these combined with the disc bulge and the aforementioned in folding of ligament and joint capsule cause pressure on the exiting nerve roots or the spinal cord itself.

The underlying mechanism of radicular pain is not clearly understood, but it is generally believed that an inflamed nerve root will produce pain when compressed. This inflammation is most likely a result of neurogenic chemical mediators released from the cell bodies of sensory neurons and nonneurogenic mediators released from the degenerate disc [20, 21]. It may also be that the dorsal root ganglion is the culprit, as it is very sensitive to deformation [22].

Myelopathy is invariably caused by compression of the spinal cord. Animal studies have demonstrated that the spinal cord must be subjected to at least 40% compression to produce reversible neurological deficits [23]. To achieve this compression ratio in patients there would usually be associated factors, such as developmental stenosis (an anteroposterior canal diameter of <13 mm) or instability of a motion segment leading to dynamic compression [24].

## 3. Treatment

### 3.1. Medications

#### 3.1.1. Non-Steroidal Anti-Inflammatory Drugs (NSAID's)

Despite an absence of clinical trials in the use of NSAIDs in the treatment of cervical spondylosis, their use is widespread. Theoretically in addition to a purely analgesic effect NSAIDs will reduce inflammation around the nerve route decreasing its sensitivity to compression. There is no evidence that NSAIDs are more effective than pure analgesics such as acetaminophen; however their efficacy has been shown in hip and knee arthritis [25, 26], and meta-analysis of the use of NSAID's in acute low back pain demonstrates greater efficacy than placebo [27–29].

#### 3.1.2. Opioid Analgesics

The use of opioid analgesics in spondylotic syndromes has been limited by the possibility of their ineffectiveness in neuropathic pain [30] in addition to the fear of their addictive nature. Despite this, there is evidence that oxycodone can be effective in the treatment of spondylosis [31, 32]. Opioid analgesics are indicated in the management of carefully selected patients with moderate to severe symptoms of axial neck pain with significant underlying structural spondylosis that are refractory to nonopioid agents and nonpharmacological therapies [33].

#### 3.1.3. Muscle Relaxants

The use of muscle relaxants in cervical spondylosis is aimed at relieving any associated spasm of the trapezius and paraspinal muscles, as well improved sleep from sedative effects of these medications. This has been demonstrated using the agent carbobenzaprine in mixed back and neck pain populations [34, 35], though the greatest gains appear to be within the first week of treatment [36]. It should be noted that because of their habit-forming properties the duration of treatment with muscle relaxants should be tapered quickly and last for a maximum of two weeks [33].

#### 3.1.4. Antidepressants

There is little evidence to support the use of antidepressants for the treatment of cervical spondylosis, but there is evidence of a modest improvement in pain severity with minimal functional improvement in low back pain [37, 38].

#### 3.1.5. Anticonvulsants

Gabapentin has been shown to improve pain in the treatment of diabetic neuropathy and has therefore become widely used for the treatment of other sources of neuropathic pain. Again there is no evidence to support its use in cervical radiculopathy, but there is evidence of small improvements in pain scores when used in lumbar radiculopathy [39, 40]. A trial of the anticonvulsant Topiramate found a small improvement in pain score but no improvement in function when used to treat lumbar radiculopathy [41]. 

#### 3.1.6. Corticosteroids

There is limited evidence to support the use of systemic corticosteroids in the treatment of cervical radiculopathy. If used the general advice is that a 1-2-week course (tapered after 3 days) of steroid such as prednisone should be used only in carefully selected patients refractory to other medication [8]. There is stronger evidence supporting the use of steroids selectively, in the form of cervical epidurals with moderate improvement of symptoms [42, 43], though cervical epidurals are not without risk [44], and a recent randomised blinded study (albeit with small numbers) has demonstrated no effect [45].

#### 3.1.7. Botulinum-A

There is moderate evidence that botulinum-A injections are of no benefit [46].

#### 3.1.8. Physiotherapy

There have been several trials and systematic reviews into the use of a structured physical therapy programme for the treatment of cervical spondylosis and its sequelae. The overall message or the prospective randomised trials appears to be that surgically treated patients receive greater improvements in pain, muscle strength, and sensory function in the early follow-up period, but at 1 year there is no difference between groups either objectively or in terms of patient satisfaction. This applies to pure axial neck pain, cervical radiculopathy [47, 48], or mild cervical myelopathy [49]. Typically the therapy regime requires 15–20 sessions of between 30- and 45- minute duration over a 3-month period. The treatment should be tailored to individual patients but includes supervised isometric exercises, proprioceptive reeducation, and manual therapy. Thermal therapy provides symptomatic relief only, and ultrasound appears to be ineffective [50].

### 3.2. Alternative Medicine

#### 3.2.1. Chinese Herbal Medicine

Chinese herbal medicine is a popular method of alternative medical therapy. The literature is sparse regarding the effectiveness of Chinese medicine for any condition, and what little literature does exist is in Chinese and is not easy to search. There is one meta-analysis that investigated the effectiveness of Chinese herbal medicine, finding only 4 studies (two of which were unpublished). This study concluded that there was weak evidence to suggest that Compound Qishe Tablet was superior to placebo and that topical Compound Extractum Nucis Vomicae was more effective than topical diclofenac gel.

#### 3.2.2. Accupuncture

Accupuncture has been shown to be effective for pain relief in the immediate and short term posttreatment period; however there is no medium to long-term benefit in pain control or functional improvement [51–54].

## 4. Conclusions

Cervical spondylosis is a common and disabling condition. The initial management should be nonoperative for the first 3 months. Surgery should be reserved for moderate to severe myelopathy patients who have failed a period of conservative treatment and patients whose symptoms are not adequately controlled by nonoperative means. Nonoperative treatment is labour intensive, requiring regular review and careful selection of medications and physical therapy on a case by case basis. More invasive treatments such as epidurals may be of benefit in a select group of patients that do not respond to simpler measures.

## References

[1] Rao RD, Currier BL, Albert TJ (2007). Degenerative cervical spondylosis: clinical syndromes, pathogenesis, and management. *Journal of Bone and Joint Surgery—Series A*.

[2] Lehto IJ, Tertti MO, Komu ME, Paajanen HEK, Tuominen J, Kormano MJ (1994). Age-related MRI changes at 0.1 T in cervical discs in asymptomatic subjects. *Neuroradiology*.

[3] Matsumoto M, Fujimura Y, Suzuki N (1998). MRI of cervical intervertebral discs in asymptomatic subjects. *Journal of Bone and Joint Surgery—Series B*.

[4] Måkelå M, Heliovaara M, Sievers K, Impivaara O, Knekt P, Aromaa A (1991). Prevalence, determinants, and consequences of chronic neck pain in Finland. *American Journal of Epidemiology*.

[5] Côté P, Cassidy JD, Carroll L (1998). The Saskatchewan health and back pain survey: the prevalence of neck pain and related disability in Saskatchewan adults. *Spine*.

[6] Urwin M, Symmons D, Allison T (1998). Estimating the burden of musculoskeletal disorders in the community: the comparative prevalence of symptoms at different anatomical sites, and the relation to social deprivation. *Annals of the Rheumatic Diseases*.

[7] Dillin W, Booth R, Cuckler J, Balderston R, Simeone F, Rothman R (1986). Cervical radiculopathy (review). *Spine*.

[8] Saal JS, Saal JA, Yurth EF (1996). Nonoperative management of herniated cervical intervertebral disc with radiculopathy. *Spine*.

[9] Ahlgren BD, Garfin SR (1996). Cervical radiculopathy. *Orthopedic Clinics of North America*.

[10] Chesnut RM, Abitbol JJ, Garfin SR (1992). Surgical management of cervical radiculopathy: indication, techniques, and results. *Orthopedic Clinics of North America*.

[11] Hurwitz EL, Carragee EJ, van der Velde G (2009). Treatment of neck pain: noninvasive interventions. Results of the bone and joint decade 2000—2010 task force on neck pain and its associated disorders. *Journal of Manipulative and Physiological Therapeutics*.

[12] Rao R (2002). Neck pain, cervical radiculopathy, and cervical myelopathy: pathophysiology, natural history, and clinical evaluation. *Journal of Bone and Joint Surgery—Series A*.

[13] Ferlic DC (1963). The nerve supply of the cervical intervertebral discs in man. *Bulletin of the Johns Hopkins Hospital*.

[14] Bogduk N, Windsor M, Inglis A (1988). The innervation of the cervical intervertebral discs. *Spine*.

[15] Grubb SA, Kelly CK (2000). Cervical discography: clinical implications from 12 years of experience. *Spine*.

[16] Dwyer A, Aprill C, Bogduk N (1990). Cervical zygapophyseal joint pain patterns I: a study in normal volunteers. *Spine*.

[17] Aprill C, Dwyer A, Bogduk N (1990). Cervical zygapophyseal joint pain patterns II: a clinical evaluation. *Spine*.

[18] Bogduk N, Marsland A (1988). The cervical zygapophysial joints as a source of neck pain. *Spine*.

[19] Dreyfuss P, Michaelsen M, Fletcher D (1994). Atlanto-occipital and lateral atlanto-axial joint pain patterns. *Spine*.

[20] Chabot MC, Montgomery DM (1995). The pathophysiology of axial and radicular neck pain. *Seminars in Spine Surgery*.

[21] Cornefjord M, Olmarker K, Farley DB, Weinstein JN, Rydevik B (1995). Neuropeptide changes in compressed spinal nerve roots. *Spine*.

[22] Rydevik BL, Myers RR, Powell HC (1989). Pressure increase in the dorsal root ganglion following mechanical compression: closed compartment syndrome in nerve roots. *Spine*.

[23] Hukuda S, Mochizuki T, Ogata M (1985). Operations for cervical spondylotic myelopathy. A comparison of the results of anterior and posterior procedures. *Journal of Bone and Joint Surgery—Series B*.

[24] Wang B, Liu H, Wang H, Zhou D (2006). Segmental instability in cervical spondylotic myelopathy with severe disc degeneration. *Spine*.

[25] Pincus T, Koch GG, Sokka T (2001). A randomized, double-blind, crossover clinical trial of diclofenac plus misoprostol versus acetaminophen in patients with osteoarthritis of the hip or knee. *Arthritis and Rheumatism*.

[26] Felson DT (2001). The verdict favors nonsteroidal antiinflammatory drugs for treatment of osteoarthritis and a plea for more evidence on other treatments. *Arthritis and Rheumatism*.

[27] Koes BW, Scholten RJPM, Mens JMA, Bouter LM (1997). Efficacy of non-steroidal anti-inflammatory drugs for low back pain: a systematic review of randomised clinical trials. *Annals of the Rheumatic Diseases*.

[28] van Tulder MW, Scholten RJPM, Koes BW, Deyo RA (2000). Nonsteroidal anti-inflammatory drugs for low back pain: a systematic review within the framework of the Cochrane Collaboration Back Review Group. *Spine*.

[29] Roelofs PDDM, Deyo RA, Koes BW, Scholten RJPM, van Tulder MW (2008). Nonsteroidal anti-inflammatory drugs for low back pain: an updated cochrane review. *Spine*.

[30] Portenoy RK, Foley KM (1986). Chronic use of opioid analgesics in non-malignant pain: report of 38 cases. *Pain*.

[31] Caldwell JR, Hale ME, Boyd RE (1999). Treatment of osteoarthritis pain with controlled release oxycodone or fixed combination oxycodone plus acetaminophen added to nonsteroidal antiinflammatory drugs: a double blind, randomized, multicenter, placebo controlled trial. *Journal of Rheumatology*.

[32] Ma K, Jiang W, Zhou Q, Du DP (2008). The efficacy of oxycodone for management of acute pain episodes in chronic neck pain patients. *International Journal of Clinical Practice*.

[33] Mazanec D, Reddy A (2007). Medical management of cervical spondylosis. *Neurosurgery*.

[34] Aker PD, Gross AR, Goldsmith CH, Peloso P (1996). Conservative management of mechanical neck pain: systematic overview and meta-analysis. *British Medical Journal*.

[35] Malanga GA, Ruoff GE, Weil AJ, Altman CA, Xie F, Borenstein DG (2009). Cyclobenzaprine ER for muscle spasm associated with low back and neck pain: two randomized, double-blind, placebo-controlled studies of identical design. *Current Medical Research and Opinion*.

[36] Browning R, Jackson JL, O’Malley PG (2001). Cyclobenzaprine and back pain: a meta-analysis. *Archives of Internal Medicine*.

[37] Hampton Atkinson J, Slater MA, Williams RA (1998). A placebo-controlled randomized clinical trial of nortriptyline for chronic low back pain. *Pain*.

[38] Salerno SM, Browning R, Jackson JL (2002). The effect of antidepressant treatment on chronic back pain: a meta-analysis. *Archives of Internal Medicine*.

[39] McCleane GJ (2001). Does gabapentin have an analgesic effect on background, movement and referred pain? A randomised, double-blind, placebo controlled study. *Pain Clinic*.

[40] Yildirim K, Sisecioglu  M, Karatay S (2003). The effectiveness of gabapentin in patients with chronic radiculopathy. *Pain Clinic*.

[41] Khoromi S, Patsalides A, Parada S, Salehi V, Meegan JM, Max MB (2005). Topiramate in chronic lumbar radicular pain. *Journal of Pain*.

[42] Manchikanti L, Cash KA, Pampati V, Wargo BW, Malla Y (2010). Cervical epidural injections in chronic-discogenic neck pain without disc herniation or radiculitis: preliminary results of a randomized, double-blind, controlled trial. *Pain Physician*.

[43] Benyamin R, Singh V, Parr AT, Conn A, Diwan S, Abdi S (2009). Systematic review of the effectiveness of cervical epidurals in the management of chronic neck pain. *Pain Physician*.

[44] Malhotra G, Abbasi A, Rhee M (2009). Complications of transforaminal cervical epidural steroid injections. *Spine*.

[45] Anderberg L, Annertz M, Persson L, Brandt L, Säveland H (2007). Transforaminal steroid injections for the treatment of cervical radiculopathy: a prospective and randomised study. *European Spine Journal*.

[46] Gross AR, Goldsmith C, Hoving JL (2007). Conservative management of mechanical neck disorders: a systematic review. *Journal of Rheumatology*.

[47] Persson LCG, Carlsson CA, Carlsson JY (1997). Long-lasting cervical radicular pain managed with surgery, physiotherapy, or a cervical collar: a prospective, randomized study. *Spine*.

[48] Persson LCG, Moritz U, Brandt L, Carlsson CA (1997). Cervical radiculopathy: pain, muscle weakness and sensory loss in patients with cervical radiculopathy treated with surgery, physiotherapy or cervical collar: a prospective, controlled study. *European Spine Journal*.

[49] Matz PG (2006). Does nonoperative management play a role in the treatment of cervical spondylotic myelopathy?. *Spine Journal*.

[50] Lee JC, Lin DT, Hong CZ (1997). The effectiveness of simultaneous thermotherapy with ultrasound and electrotherapy with combined AC and DC current on the immediate pain relief of myofascial trigger points. *Journal of Musculoskeletal Pain*.

[51] Petrie JP, Hazleman BL (1986). A controlled study of acupuncture in neck pain. *British Journal of Rheumatology*.

[52] Irnich D, Behrens N, Molzen H (2001). Randomised trial of acupuncture compared with conventional massage and “sham” laser acupuncture for treatment of chronic neck pain. *British Medical Journal*.

[53] White P, Lewith G, Prescott P, Conway J (2004). Acupuncture versus placebo for the treatment of chronic mechanical neck pain. A randomized, controlled trial. *Annals of Internal Medicine*.

[54] Coan RM, Wong G, Coan PL (1981). The acupuncture treatment of neck pain: a randomized controlled study. *American Journal of Chinese Medicine*.

